# The Research Progress of Vascular Macrophages and Atherosclerosis

**DOI:** 10.1155/2020/7308736

**Published:** 2020-05-24

**Authors:** Yeqing Tong, Li Cai, Shiyu Yang, Shuang Liu, Zhihong Wang, Jinquan Cheng

**Affiliations:** ^1^Hubei Center for Disease Control and Prevention, 430079, China; ^2^Wuhan Center for Disease Control and Prevention, Wuhan 430015, China; ^3^School of Health Sciences, Wuhan University, Wuhan 430071, China; ^4^Department of Neurology, Shenzhen No. 2 People's Hospital, The First Affiliated Hospital of Shenzhen University, Shenzhen 518035, China; ^5^Key Laboratory of Molecular Biology of Guangdong Province, Center for Disease Control and Prevention, Shenzhen 518055, China

## Abstract

**Materials:**

We made a minireview on the association of vascular macrophages with AS based on recent research studies systematically, from the mechanisms of macrophages accumulating in the walls of blood vessels, and the role of macrophages in AS as well as microenvironmental determinants of macrophage function in AS. The discovery of these mechanisms could reveal the pathogenesis of AS comprehensively and is crucial to provide scientific evidence for formulating the related measures of prevention and treatment for AS. *Discussion*. Vascular macrophages play important roles in the development of AS, and the vascular macrophages may become new targets for the prevention and treatment of AS. Effective regulation of host genes may help prevent or even treat AS.

**Conclusion:**

This minireview focuses on the association of vascular macrophages with the development of AS, which may supply some clues for future therapies and novel drug targets for AS.

## 1. Introduction

Atherosclerosis (AS) is a chronic inflammatory vascular wall disease. Its complications, especially myocardial infarction and stroke, are the most common cause of death worldwide. Macrophages are the most abundant type of immune cells in atherosclerotic lesions and play important roles in all stages of disease, from atherosclerotic lesion formation to plaque rupture [1, 2].

The role of macrophages in AS can be briefly described as follows: when the local blood flow is nonlaminar, vascular endothelial dysfunction causes circulating low-density lipoprotein (LDL) to penetrate into the lining of blood vessels, especially in hyperlipidemia. In vascular walls, LDL undergo oxidative modifications, activating endothelial cells and resident immune cells, leading to the upregulation of chemokines and adhesion molecule expression that attract circulating monocytes to vascular walls, allowing them to adhere and roll, and transendothelial migration, resulting in intimal infiltration of monocytes. After monocytes enter the intima of blood vessels, they differentiate into macrophages and take in modified lipoproteins to further become foam cells. The accumulated foam cell apoptosis in the endothelium is not cleared in time, which gradually leads to the formation of thrombus and inflammatory necrosis core [3]. Macrophages further aggravate pathological inflammation by secreting cytokines and proteolytic enzymes, leading to decreased plaque stability and rupture, forming atherosclerotic thrombosis.

### 1.1. The Mechanisms of How Macrophages Were Accumulated in the Walls of Blood Vessels

For a long time, bone marrow hematopoiesis has been considered the main source of monocytes in the development of AS, hyperlipidemia, hyperglycemia, and other AS-associated diseases [1–4]. More than 20 years ago, the proliferation of macrophages in atherosclerotic plaques was observed in human and animal models, but the quantitative contribution of macrophage accumulation to the development of lesions has not yet been clearly studied [5]. Through the distinction between sources of tissue cells, either they are tissue circulating monocyte or local expansion of the cells, macrophage proliferation was considered to account for about 87% of macrophage accumulation in established lesions, while the circulating monocytes added only works in the early stages of the disease [6]. However, data from the same study using the long-term chimeric model showed that all established macrophages in lesions ultimately came from circulating precursors and that monocytes increased with the addition proportion in the progression of lesions [7]. A recent report showed that compared to previous studies, the proliferative activity of macrophages was higher in early rather than late lesions [8]. However, it has recently been found that a large proportion of macrophages in identified lesions are senescent [9], so macrophages cannot proliferate according to the definition of cell proliferation. Moreover, studies have shown that highly proliferative and plastic vascular smooth muscle cells can present a macrophage-like phenotype and express classic macrophage markers in lesions, such as MAC 3 or MAC 2, which could further complicate the interpretation of studies on macrophage proliferation in AS [10].

In the past 20 years, markers coexpressed by macrophages and vascular smooth muscle cells (VSMC) were found in AS. A recent study showed that up to 50% of cells identified as macrophage foam cells coexpressed smooth muscle cell markers [11]. However, these studies based on immunohistological analysis were unable to determine whether the cells were derived from macrophages with upregulated VSMCs or were transformed into macrophage foam cells. In the study of pedigree fate of VSMC in a mouse model with AS, the transformation of VSMC into macrophage-like cells in lesions was confirmed [12]. In in vivo studies, VSMC-derived macrophages can form foam cells, but these cells are different from real macrophages in function [13].

Macrophages are widely expressed in humans, so healthy arteries already contain resident macrophages and dendritic cells that may self-renew after infection-induced failure. The role of macrophages, which are present in the arteries by themselves, remains to be determined in AS, but some studies have suggested that a group of epigastric macrophage progenitors exist in the aorta of adult mice and may locally differentiate to promote the accumulation of macrophages [14].

**Figure 1:**
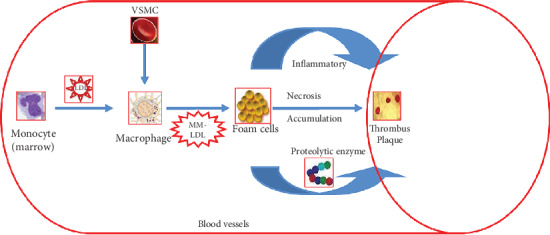
The mechanisms of how macrophages were accumulated in the walls of blood vessels; VSMC: vascular smooth muscle cell; MM-LDL: minimally modified low-density lipoprotein.

Atherosclerotic plaque and its macrophage content can be decreased, which has been shown in a hypercholesterolemia reversal mouse model [15]. However, the exact potential mechanism is still controversial, and some studies have suggested that macrophage autophagy may be the cause of this phenomenon [16]. Some reports of atherosclerotic aortic transplantation models have found that pathological changes in mature macrophages during migration will lead to the formation of plaques [17]. Another study showed that the reduced load of macrophages is not related to macrophage migration but depends on the inhibition of monocyte accumulation and the apoptosis of plaque macrophages [15]; the mechanisms of how macrophages were accumulated in the walls of blood vessels are described in Figure 1.

### 1.2. The Role of Macrophages in AS

After entering into the lesion site, macrophages actively participate in vascular inflammation by secreting proinflammatory cytokines and producing chemokines, further promoting absorption of immune cells. Plaque macrophages express proinflammatory cytokines, such as tumor necrosis factor alpha (TNF alpha), interleukin-18 (IL-18), and interleukin-12 (IL-12) [4]. CCL2 overexpression in bone marrow cells increases macrophages' absorption of lesions, suggesting that leukocyte derived from chemokines can fuel plaque inflammation [18]. Meanwhile, macrophages were known to have a strong proteolysis activity and were considered to be related to the instability and rupture of plaques [19].

The formation of macrophage-derived foam cells in the intima of blood vessels is a hallmark of AS. Macrophages ingest modified LDL through a variety of scavenger receptors, such as CD36, scavenger receptor A1 (sr-a1, also known as MSR-1) [20], scavenger receptor B1, LDL receptor-related protein 1 (LRP-1), and lectin-like receptor 1 (LOX-1) [21, 22]. Several studies have shown that foam cell formation reduced in mice lacking specificity in MSR-1 [23]. In addition to its role in lipid uptake, MSR-1 was also found to play a key role in the proliferation of macrophages in advanced lesions [6].

Given the importance of macrophage proliferation in macrophage infiltration, the proliferative ability can also be considered an important functional feature of disease-associated macrophages. Since macrophage proliferation is preferentially activated in advanced lesions [10]. Deciphering the mechanism of transition from a nonproliferative state to a proliferative state is worth studying. Compared to proliferating macrophages, the lesion also contains senescent cells that have lost their ability to proliferate. Although the pathogenic role of senescent cells in AS has been suspected [23], a recent report pointed out that foam cells of senescent macrophages in the intima of blood vessels were pathogenic in the whole process of AS [24]. In conclusion, the increase of macrophage survival or apoptosis ultimately determines the progression and regression of plaque, which is likely to depend on the stability of plaque macrophages and aggregation of monocytes [16].

### 1.3. Microenvironmental Determinants of Macrophage Function in AS

The effective cellular function of apoptotic cells can also promote the accumulation of intracellular lipids in macrophages, and how this affects the macrophage phenotype remains unclear [18]. Cholesterol crystals can form both intracellular and extracellular lesions, and cholesterol crystal deposits can be detected even in the early stage of atherosclerotic lesions. It is worth noting that cholesterol crystals activate NLRP-3 inflammasome which further transforms proinflammatory cytokines IL-1 beta and IL-18 into mature and secretory forms and converts them into cholesterol crystal mediators. Activation of NLRP-3 inflammasome has been considered essential in the development of AS [25].

In the late stage of AS, the increased distance from the vascular lumen to the plaque core increases the metabolic demands, leading to hypoxic areas. Hypoxia induces the expression of oxygen-sensitive transcription factor-1 alpha (HIF1 alpha), a process that can be further demonstrated by inflammatory signaling pathways such as NF-kappa B [26]. Therefore, hypoxia may be involved in the development of AS.

Macrophages are also activated by growth factors, such as granulocyte-macrophage colony-stimulating factor (CSF-2) or CSF-1, which control macrophage survival and proliferation. CSF-2 also indirectly induces apoptosis of plaque macrophages by upregulating the expression of IL-9 [27]. The plaque microenvironment also contains a large number of proinflammatory and anti-inflammatory cytokines that affect the macrophage phenotype. Cytokines such as IL-1 beta, TNF alpha, or IFN-gamma promote the formation of proatherogenic macrophage phenotype and increase the expression of proinflammatory cytokines, chemokines, and proteases [28, 29].

## 2. Conclusions

Taken together, these studies supported that vascular macrophages play important roles in mediating the regulation of AS infection. The development of AS is correlated with vascular macrophages, and vascular macrophage plays a unique role in the development of AS. But most effects in different subjects are inconsistent, which may be influenced by a variety of factors. Therefore, it is necessary to conduct repetitive, multicenter, and large-sample researches in order to draw more scientific conclusions, to further confirm the relationship between vascular macrophages and AS, and to better clarify the immunopathological mechanisms of AS for a theoretical basis for prevention and treatment as well as to provide clues for finding new drug targets for AS-associated diseases.
